# Association of vitamin D receptor *Fok I* polymorphism with the risk of prostate cancer: a meta-analysis

**DOI:** 10.18632/oncotarget.12837

**Published:** 2016-10-24

**Authors:** Shaosan Kang, Yansheng Zhao, Jian Liu, Lei Wang, Geng Zhao, Xi Chen, Anliang Yao, Liguo Zhang, Xiaojun Zhang, Xiaoqiang Li

**Affiliations:** ^1^ Department of Urology, North China University of Science and Technology Affiliated Hospital, Tangshan 063000, China; ^2^ Department of Imaging, KaiLuan General Hosptial, Tangshan 063000, China; ^3^ Department of Urology, KaiLuan General Hospital, Tangshan 063000, China

**Keywords:** Fok I, prostate cancer, vitamin D receptor, polymorphisms, meta-analysis

## Abstract

Several previous studies have been reported to examine the association between Vitamin D receptor (*VDR*) gene *Fok I* polymorphism and susceptibility to prostate cancer (PCa), however the results remain inconclusive. To provide a relatively comprehensive account of the association, we searched PubMed, Embase, CNKI, and Wanfang for eligible studies and carry out this meta-analysis. A total of 27 case-control studies with 10,486 cases and 10,400 controls were included. In the overall analysis, *Fok I* polymorphism was not significantly associated with the susceptibility to PCa. Subgroup analyses showed that significantly association was existed in Caucasian population, the subgroup of population-based controls and the stratified group with advanced tumor.These results indicate that the *VDR Fok I* polymorphism might be capable of causing PCa susceptibility and could be a promising target to forecast the PCa risk for clinical practice. However further well-designed epidemiologic studies are needed to confirm this conclusion.

## INTRODUCTION

Prostate cancer (PCa) is now thought to be one of the most commonly diagnosed malignant tumors in old men throughout the world, and the second cause of cancer in males. It accounted for approximately 233,000 (27%) new cases and 30,000 deaths in the United States in 2014 [1]. The global incidence of PCa has increased annually. The etiology of PCa is largely unknown. Several factors have been suggested to be strongly associated with the increased risk, including ethnic origin, family history, hormonal status, dietary structure and age [2].

Low levels of vitamin D are considered to be a risk factor for PCa [3]. *In vitro* experiments suggested that vitamin D inhabits the growth and differentiation of prostate cancer cells, promotes cell apoptosis. It can also inhabit the invasion, metabolism and angiogenesis of tumor cell [3]. A clinical trial of PCa patients showed that calcitriol, analogue of vitamin D can significantly reduce the prostate specical antigen (PSA) level, and improve the patients survival rate [4].

The anticancer effect of vitamin D is activated mainly through the vitamin D receptor (*VDR*) [5]. 1,25-Dihydroxy vitamin D3 (1,25(OH)_2_D_3_), the active form of vitamin D, binds to *VDR* and form a heterodimer complex, which subsequently binds to the vitamin D response element and down-regulate the transcription of numerous genes that stimulating the cell growth and differentiation [6].

Several single nucleotide polymorphisms (SNPs) of *VDR* gene were reported to be associated with risk of PCa [7]. *Fok I* variant (rs10735810) located in exon 2 of *VDR* gene is one of the most extensively studied SNPs [8]. It could result in a frame-shift mutation in the expression of VDR. It has been reported that f allele results in three amino acids longer VDR than the F allele, and extensive researches indicate that f allele is less effective than the F allele in transcription activity and transactivation of the 1,25(OH)_2_D_3_ signal [8]. Recent studies have shown that *Fok I* polymorphism might accelerate the progression of PCa. However, the results are disputable and contradictory [9, 10], as it might be underpowered for individual study. Therefore, we performed this meta-analysis to draw a more precise conclusion based on the published literature.

## RESULTS

### Characteristics of studies included in this meta-analysis

**Figure 1:**
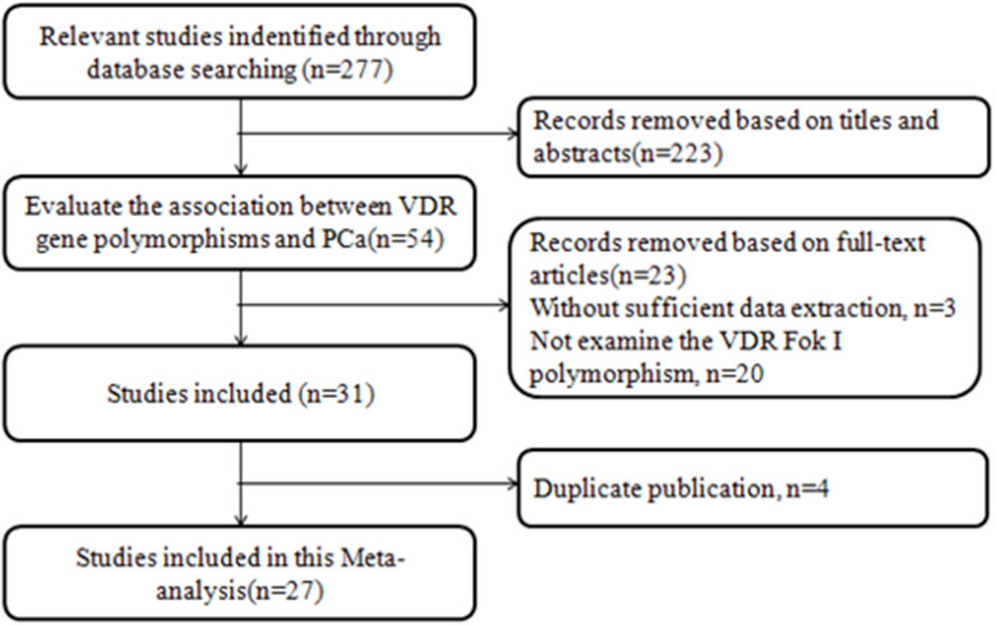
Study flowchart for the process of selecting the final 27 studies

A total of 277 potentially relevant studies were identified following the searching strategy. 27 studies [2, 6, 7, 9, 10, 12-32] were finally included in this meta-analysis according to the inclusion criteria (Figure 1). Publication years ranged from 1999 to 2015, the number of cases varied from 28 to 1,518, and the number of controls varied from 56 to 1,432 (Table 1). The distribution of genotype frequency in the control groups was in accordance with the HWE for almost studies, except two studies [9, 15. in which source of controls was hospital-based. As a result, data for our meta-analysis were available from 27 studies with a total of 10,468 cases and 10,400 controls. The eligible studies were assessed by the NOS. Each of the studies scored morethan 4, which suggested that all of them are of high quality researches (Table 1).

**Table 1:** Characteristics and quality assessment of the studies included in this meta-analysis

Study ID	Year	Country	Ethnicity	Genotyping method	Source of controls	Total sample size (case/control)	HWE	Quality indicators from NOS
Atoum	2015	Jordan	Asian	TaqMan	PB	124/100	Y	6
Bai	2009	China	Asian	PCR-RFLP	HB	122/130	Y	6
Bodiwala	2004	UK	Caucasian	PCR-RFLP	HB/BPH	368/243	Y	6
Chen	2001	China	Asian	PCR-RFLP	HB	101/145	N	5
Cheteri	2004	USA	Caucasian	PCR-RFLP	PB	552/521	Y	6
Chokkalingam	2001	China	Asian	PCR-RFLP	PB	187/302	Y	6
Cicek	2006	USA	Mixed	PCR-RFLP	PB	439/479	Y	6
Correa-Cerro	1999	Germany/France	Caucasian	PCR-RFLP	HB	118/89	Y	6
Hayes	2005	Australia	Caucasian	DGGE*	PB	811/713	Y	7
Holick	2007	USA	Caucasian	SNPlex	PB	583/552	Y	6
Holt	2009	USA	Caucasian	SNPlex	PB	705/716	Y	6
Huang	2006	China	Asian	PCR-RFLP	HB/BPH	416/502	Y	6
Jiang	2013	China	Asian	PCR-RFLP	PB	100/108	Y	6
John	2005	USA	Caucasian	TaqMan	PB	425/437	Y	6
Li	2007	USA	Caucasian	PCR-RFLP	PB	1010/1432	Y	8
Luscombe	2001	UK	Caucasian	PCR-RFLP	BPH	209/154	Y	6
Mikhak	2007	USA	Caucasian	TaqMan	PB	670/673	Y	7
Mishra	2005	India	Asian	PCR-RFLP	HB	147/128	Y	6
Oakley-Grivan	2004	USA	Mixed	PCR-RFLP	PB	345/292	Y	6
Oh	2013	Korea	Asian	IGGGS#	BPH	272/173	Y	6
Rowland	2013	USA	Mixed	TaqMan	PB	1518/1070	Y	7
Ruan	2009	China	Asian	PCR-RFLP	BPH	100/100	Y	5
Rukin	2007	UK	Caucasian	Pyrosequencing	BPH	430/320	Y	6
Tayeb	2004	UK	Caucasian	PCR-RFLP	BPH	28/56	Y	6
Torkko	2008	USA	Caucasian	TaqMan	PB	585/761	Y	6
Yang	2004	China	Asian	PCR-RFLP	PB	80/96	Y	5
Yousaf	2014	Pakistani	Asian	PCR-RFLP	HB	41/108	N	6

Abbreviations: HWE, Hardy-Weinberg equilibrium; PB, population-based; HB, hospital-based; BPH, Benign Prostate Hyperplasia; RFLP, restriction fragment length polymorphism; DGGE, denaturing gradient gel electrophoresis; IGGGS, Illumina Golden Gate genotyping system

### Meta-analysis results

**Figure 2:**
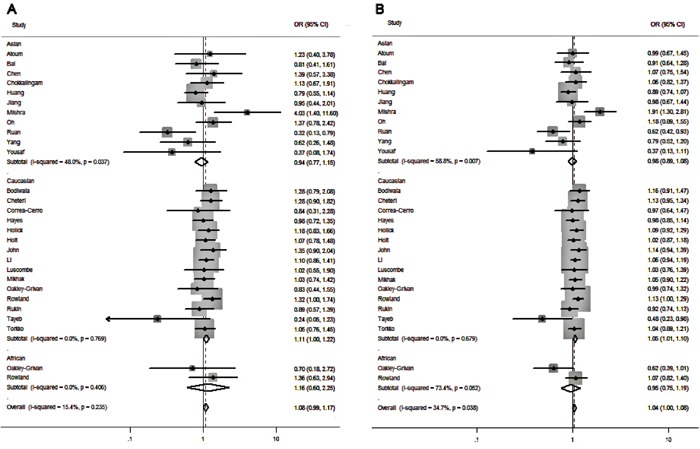
Forest plots to estimate the association of VDR Fok I polymorphism with PCa in the subgroup analysis of ethnicity **A.** Homozygote model (ff vs. FF). **B.** Allelic frequency model (f vs. F allele).

The results of overall analysis are showed in Table 2 and Figure 2. The pooled results indicated that *Fok I* polymorphism is not associated with the PCa risk in the overall populations (ff vs. FF: OR=1.07, 95%CI=0.98-1.16, p=0.131; Ff vs. FF: OR=1.03, 95%CI=0.97-1.10, p=1.05; Ff/ff vs. FF: OR= 1.04, 95%CI= 0.98-1.10, p=0.173; ff vs. FF/Ff: OR=1.04, 95%CI=0.96-1.12, p=0.318; f vs. F allele: OR=1.03, 95%CI=0.99-1.07, p=0.138). (Table 2).

**Table 2:** Results of the association between *Fok I* polymorphism and PCa risk in the whole population

Comparison	Studies	Overall effect			Heterogeneity		Public bias	
		OR	Z-score	p-value	I^2^	P-value	Begg's test	Egger's test
**ff vs FF**	27	1.07 [0.98-1.16]	1.51	0.131	14%	0.255	0.087	0.118
**Ff vs FF**	27	1.03 [0.97-1.10]	1.05	0.296	0%	0.809	0.402	0.866
**ff+Ff vs FF**	27	1.04 [0.98-1.10]	1.36	0.173	0%	0.475	0.133	0.322
**ff vs FF+Ff**	27	1.04 [0.96-1.12]	1	0.318	13%	0.274	0.227	0.138
**f vs F**	27	1.03 [0.99-1.07]	1.48	0.138	27%	0.102	0.027	0.101

For the subgroup analysis of ethnicity stratification. Significantly increased risk of PCa was detected in Caucasian populations in the comparison of homozygote model (ff vs. FF: OR=1.107, 95%CI=1.005-1.219, p=0.04), dominant model (Ff/ff vs. FF: OR=1.079, 95%CI=1.010-1.152, p=0.024) and allele-frequency genetic model (f vs. F allele: OR=1.054, 95%CI=1.006-1.103, p=0.026)(Table 3 & Figure 2). However, when 11 studies conducted in Asian populations and 2 studies in African populations were analyzed, no significant associations were found between *Fok I* polymorphism and the susceptibility to PCa (Table 3).

**Table 3:** Results of the association between *Fok I* polymorphism and PCa risk in different ethnicities

Comparison	Studies	Overall effect			Heterogeneity		Public bias	
		OR	Z-score	p-value	*I*^2^	P-value	Begg's test	Egger's test
**Asian**								
ff vs FF	11	0.940 [0.771-1.150]	0.58	0.561	48%	0.037	0.876	0.901
Ff vs FF	11	1.032 [0.880-1.210]	0.39	0.696	18%	0.276	0.721	0.819
Ff/ff vs FF	11	1.003 [0.864-1.166]	0.04	0.964	43%	0.063	0.213	0.635
ff vs FF/Ff	11	0.944 [0.797-1.117]	0.67	0.501	41%	0.078	0.876	0.95
f vs F	11	0.983 [0.892-1.082]	0.36	0.722	59%	0.007	0.213	0.637
**Caucasian**								
ff vs FF	15	1.107 [1.005-1.219]	2.06	0.04	0%	0.769	0.138	0.034
Ff vs FF	15	1.070 [0.998-1.147]	1.9	0.058	0%	0.973	0.488	0.562
Ff/ff vs FF	15	1.079 [1.010-1.152]	2.25	0.024	0%	0.915	0.488	0.176
ff vs FF/Ff	15	1.057 [0.969-1.152]	1.24	0.214	0%	0.694	0.276	0.089
f vs F	15	1.054 [1.006-1.103]	2.23	0.026	0%	0.679	0.428	0.06
**African**								
ff vs FF	2	1.165 [0.603-2.249]	0.45	0.65	0%	0.406	1	-
Ff vs FF	2	0.861 [0.646-1.148]	1.02	0.309	73%	0.055	1	-
Ff/ff vs FF	2	0.899 [0.673-1.173]	0.83	0.405	75%	0.045	1	-
ff vs FF/Ff	2	1.215 [0.633-2.330]	0.58	0.559	0%	0.554	1	-
f vs F	2	0.945 [0.751-1.189]	0.48	0.631	73%	0.052	1	-

**Figure 3:**
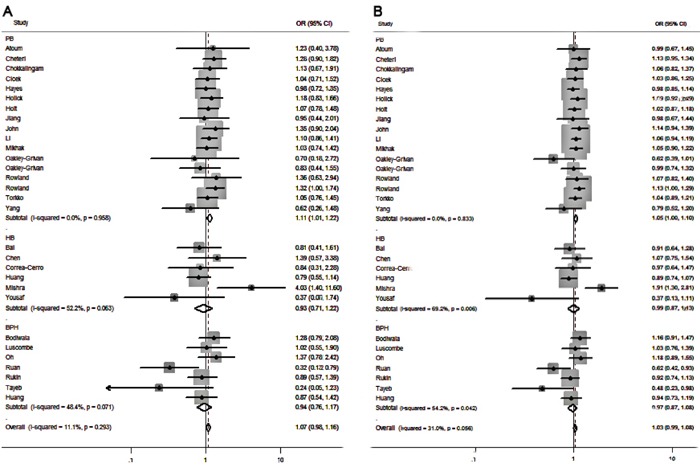
Forest plots to estimate the association of VDR Fok I polymorphism with PCa in the subgroup analysis of source of controls **A.** Homozygote model (ff vs. FF). **B.** Allelic frequency model (f vs. F allele).

For the stratified analysis of source of controls. We found that *Fok I* polymorphism could significantly increase the risk of PCa in the subgroup of population-based controls in homozygote model (ff vs. FF: OR=1.112, 95%CI=1.011-1.223, p=0.029) and allele-frequency genetic model (f vs. F allele: OR=1.005-1.099, p=0.03) (Table 4 & Figure 3). Meanwhile, no significantly increased risk was observed in the subgroups of hospital-based or BPH controls (Table 4).

**Table 4:** Results of the association between *Fok I* polymorphism and PCa risk in different source of controls

Comparison	Studies	Overall effect			Heterogeneity		Public bias	
		OR	Z-score	p-value	*I*^2^	P-value	Begg's test	Egger's test
**Population-based**								
ff vs FF	15	**1.112 [1.011-1.223]**	2.19	0.029	0%	0.958	0.434	0.186
Ff vs FF	15	1.051[0.983-1.124]	1.45	0.148	0%	0.809	0.202	0.126
Ff/ff vs FF	15	1.064 [0.998-1.133]	1.9	0.058	0%	0.811	0.174	0.053
ff vs FF/Ff	15	1.074 [0.984-1.171]	1.6	0.109	0%	0.935	0.773	0.367
f vs F	15	**1.051 [1.005-1.099]**	2.17	0.03	0%	0.833	1.108	0.016
**Hospital-based**								
ff vs FF	6	0.931 [0.711-1.219]	0.52	0.062	52%	0.063	0.452	0.524
Ff vs FF	5	1.088 [0.866-1.337]	0.81	0.42	47%	0.11	0.806	0.419
Ff/ff vs FF	6	1.045 [0.862-1.268]	0.45	0.653	59%	0.033	0.452	0.999
ff vs FF/Ff	6	0.910 [0.718-1.152]	0.79	0.432	46%	0.103	1	0.642
f vs F	6	0.992 [0.871-1.129]	0.13	0.897	69%	0.006	1	0.973
**BPH**								
ff vs FF	7	0.941 [0.982-1.159]	0.55	0.584	48%	0.071	0.548	0.077
Ff vs FF	7	1.030 [0.861-1.231]	0.32	0.748	0%	0.678	0.23	0.025
Ff/ff vs FF	7	1.001 [0.846-1.183]	0.01	0.994	26%	0.231	0.368	0.037
ff vs FF/Ff	7	0.928 [0.955-1.107]	0.85	0.394	35%	0.159	0.368	0.196
f vs F	7	0.972 [0.875-1.081]	0.52	0.604	54%	0.042	0.368	0.102

In the stratified analysis by genotyping method, there was no significant association in different subgroups, which were stratified into TaqMan, PCR-RFLP, SNPlex and other subgroups. As showed in Table 5, the pooled outcome showed that the genotyping methods reported in the included studies are both effective and applicative. Among the 27 studies included in our meta-analysis, there were two studies that deviated from HWE in the controls [9], we conducted a subgroup analysis. When the 2 studies excluded, another result obtained, which is similar to the overall analysis (The result was not given).

**Table 5:** Results of the association between *Fok I* polymorphism and PCa risk in different genotyping method

Comparison	Studies	Overall effect			Heterogeneity		Public bias	
		OR	Z-score	p-value	*I*^2^	P-value	Begg's test	Egger's test
**PCR-RFLP**								
ff vs FF	17	1.014 [0.895-1.148]	0.21	0.83	36%	0.068	0.077	0.182
Ff vs FF	16	1.063 [0.970-1.165]	1.3	0.192	0%	0.611	0.192	0.565
Ff/ff vs FF	17	1.051 [0.964-1.146]	1.13	0.257	27%	0.149	0.053	0.18
ff vs FF/Ff	17	0.983 [0.822-1.189]	0.3	0.766	23%	0.188	0.149	0.176
f vs F	17	1.020 [0.960-1.083]	0.63	0.526	49%	0.012	0.019	0.127
**TaqMan**								
ff vs FF	5	1.155 [0.989-1.349]	1.82	0.068	0%	0.8	1	0.822
Ff vs FF	5	1.018 [0.914-1.134]	0.33	0.74	8%	0.364	0.806	0.785
Ff/ff vs FF	5	1.047 [0.946-1.159]	0.88	0.377	0%	0.676	1	0.854
ff vs FF/Ff	5	1.131 [0.981-1.305]	1.69	0.09	4%	0.385	0.806	0.891
f vs F	5	1.056 [0.983-1.136]	1.49	0.137	0%	0.934	0.806	0.989
**SNPlex**								
ff vs FF	2	1.120 [0.866-1.416]	0.95	0.343	0.00%	0.702	1	-
Ff vs FF	2	1.003 [0.846-1.188]	0.03	0.976	0%	0.532	1	-
Ff/ff vs FF	2	1.031 [0.983-1.102]	0.37	0.712	0.00%	0.509	1	-
ff vs FF/Ff	2	1.118 [0.902-1.386]	1.02	0.309	0.00%	0.884	1	-
f vs F	2	1.047 [0.935-1.171]	1.48	0.138	0%	0.57	1	-
**Others**								
ff vs FF	3	1.013 [0.802-1.280]	0.11	0.913	0%	0.475	1	0.607
Ff vs FF	3	0.995 [0.828-1.195]	0.06	0.956	0%	0.803	0.296	0.175
Ff/ff vs FF	3	0.994 [0.837-1.182]	0.06	0.95	0%	0.656	0.296	0.49
ff vs FF/Ff	3	0.989 [0.822-1.189]	0.12	0.904	1%	0.365	1	0.362
f vs F	3	0.944 [0.889-1.110]	0.11	0.91	1%	0.366	1	0.637

**Figure 4:**
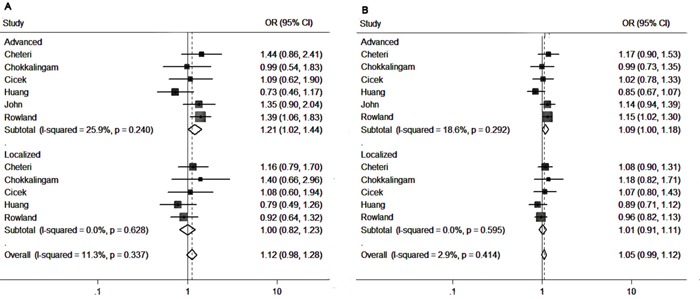
Forest plots to estimate the association of VDR Fok I polymorphism with PCa in the subgroup analysis of tumor stage **A.** Homozygote model (ff vs. FF). **B.** Allelic frequency model (f vs. F allele).

A subgroup analysis based on the tumor stages was also conducted to delineate the association in more detail. As presented in Table 6 and Figure 4, the pooled results from 6 studies showed that *Fok I* polymorphism is associated with the advanced tumor in homozygote model (ff vs. FF: OR=1.210, 95%CI=1.020-1.437, p=0.029) and allele-frequency genetic model (f vs. F allele: OR=1.085, 95%CI=1.000-1.178, p=0.05). Meanwhile, no significant difference in the genetic variants was detected between localized tumor cases or controls.

**Table 6:** Results of the association between *Fok I* polymorphism and PCa risk in different tumor stage

Comparison	Studies	Overall effect			Heterogeneity		Public bias	
		OR	Z-score	p-value	*I*^2^	P-value	Begg's test	Egger's test
**Advanced**								
ff vs FF	6	**1.210 [1.020-1.437]**	2.18	0.029	26%	0.24	0.26	0.278
Ff vs FF	6	1.023 [0.904-1.158]	0.36	0.715	0%	0.832	0.707	0.112
Ff/ff vs FF	6	1.070 [0.952-1.202]	1.13	0.259	0%	0.564	0.452	0.164
ff vs FF/Ff	6	1.194 [1.022-1.395]	2.23	0.026	5%	0.388	0.26	0.412
f vs F	6	**1.085 [1.000-1.178]**	1.96	0.05	19%	0.292	0.26	0.271
**Localized**								
ff vs FF	5	1.002 [0.817-1.229]	0.02	0.984	0%	0.628	0.462	0.482
Ff vs FF	5	1.031 [0.891-1.193]	0.41	0.679	0%	0.902	0.462	0.28
Ff/ff vs FF	5	1.024 [0.892-1.175]	0.34	0.737	0%	0.768	0.462	0.384
ff vs FF/Ff	5	0.980 [0.814-1.179]	0.22	0.828	0%	0.731	0.462	0.512
f vs F	5	1.006 [0.913-1.108]	0.12	0.903	0%	0.595	0.806	0.437

### Heterogeneity

There was no significant between-study heterogeneity in all the comparison models in the overall analysis (ff vs. FF: p=0.131, I^2^=14%), Ff vs. FF: p=0.105, I^2^=0%; Ff/ff vs. FF: p=0.173, I^2^=0%; ff vs. FF/Ff: p=0.318, I^2^=13%; and f vs. F allele: p=0.138, I^2^=27%) (Table 2). Thus, fixed-effects estimates would be more appropriate for data analysis.

### Publication bias and sensitivity analysis

**Figure 5:**
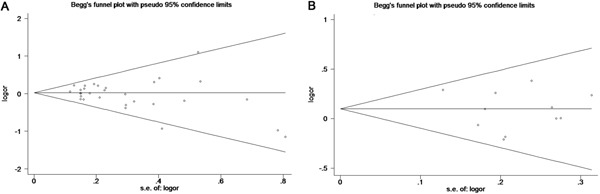
Begg's funnel plots to examine piblishcation bias for reported comparisons of *VDR* gene Fok I polymorphism **A.** Overall comparison for the recessive model (ff vs. FF/Ff). **B.** Subgroup analysis of tumor stage for the recessive model (ff vs. FF/Ff).

**Figure 6:**
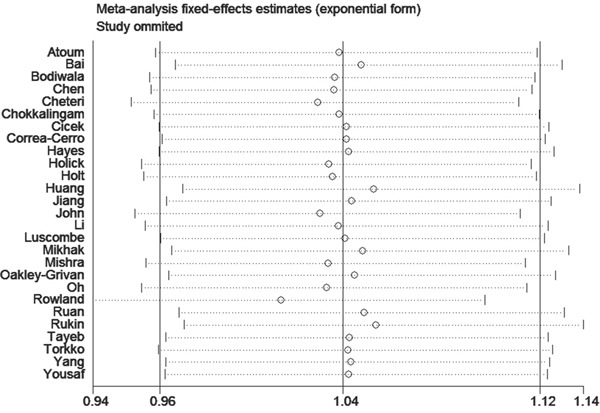
Sensitivity analysis of the comparison in recessive model (ff vs. FF/Ff) in the overall analysis

The publication bias of literature assessed with both funnel plots and Egger's test. As shown in Figure 5, it did not reveal any obvious asymmetry in the funnel plots (Figure 5). Moreover, the Egger's test which was used to provide statistical evidence of publication bias suggested that no evidence of publication bias existed in the overall analysis (p=0.118 for ff vs. FF; p=0.866 for Ff vs. FF; p=0.322 for Ff/ff vs. FF; p=0.138 for ff vs. FF/Ff; and p=0.101 for f vs. F allele) (Table 2) and almost the subgroup analyses (Table 3-6). Sensitivity analyses showed that omitting individual study from all the analyses did not affect the pooled ORs significantly, no substantial change was detected, indicating that our results were statistically robust (Figure 6).

## DISCUSSION

The *VDR* gene has earned special attention because an increasing number of studies have revealed that polymorphisms of the *VDR* gene were associated with the risk of PCa [33]. However, the results across studies have been equivocal [34, 35, 36]. Previous meta-analyses were performed by Xu et al. in 2014, Guo et al. in 2013 and Yin et al. in 2009 [34, 37, 44]. Xu et al. and Yin et al. reported the relationship of cancer risk with several *VDR* SNPs including *Fok I*. For the association of *Fok I* polymorphism with PCa, they included 19 studies and 16 studies, respectively. The shortage of these two studies is that they only performed overall analyses without any detailed subgroup analyses. Guo et al. included 22 stuides and conducted the stratified analyses. But from 2013 to now, some new data appearred, differently from the results of previous meta-analyses [34, 37, 44]. Our study included 10,468 cases and 10,400 controls from 27 independent studies, which is much more than the former three studies. Therefore, the results we obtained might be more stringent and comprehensive.

Our meta-analysis indicated the relationship of *VDR* gene *Fok I* polymorphism with the PCa risk is not existed in overall population. It is consistent with the results of previous meta-analyses [34, 37, 44]. But for the subgroup analysis of ethnicity, significant association was found in Caucasians. It is not reported by previous meta-analyses [34, 37, 44]. It suggests that in individuals of Caucasian ethnicity but not of Asians or Africans, the FF genotype and F allele might be protective. Ethnicity is one of the most important biological factors that might influence the function of VDR through gene-gene interaction [38]. The difference might be caused by the discrepancies in racial backgrounds and geography [40]. Besides, different diet structure could play a role in the discrepancies [41]. Our results suggested that the *Fok I* polymorphism could be a potential biomarker to forecast the PCa risk of Caucasians for clinical practice. Further studies of Asian and African are required.

For the source of controls, borderline significant association was found in population-based controls. Possibly some sick population were enrolled in the groups of hospital-based controls and HBP controls, so that these groups could not represent all population [42]. Hence, the results of these groups would be lack of credibility. Our results showed that no difference between the genotyping methods. It suggested that all the genotyping methods applied in the included studies are appropriate to get accurate genotype distribution. As a research reported in 2004, polymorphism would be associated with the tumor stage of PCa [43]. We also performed a stratified analysis by tumor stage. Differently from the previous meta-analyses [44, 45], we found that in the subgroup of advanced tumor stage, ff genotype and f allele might increase the PCa risk. It indicating that *Fok I* polymorphism could indeed be a risk factor associated with PCa progression.

The heterogeneity between the studies was very low in the overall analysis. It suggested that the results from these studies were suitable to be pooled [46]. Although evidence of heterogeneity existed in some subgroup analyses, the sensitivity analysis indicated that studies contribute to the heterogeneity did not significantly alter the pooled results. It suggested our results were statistically robust.

Several limitations in our meta-analysis should be acknowledged. First, several studies with small sample size included in our analysis might be underpowered to detect the relationship. Second, our results were according to the unadjusted parameters, a more accurate analysis should be performed, in which the outcomes would be adjusted by some related parameters, including age, dietary status, and other important lifestyle factors.

In conclusion, our meta-analysis might be the largest meta-analysis to estimate the association of *VDR* gene *Fok I* polymorphism with the risk of PCa. Although no significantly association of *Fok I* polymorphism with PCa risk was found in overall population, the possibility of an association in specific subpopulations such as Caucasians and the advanced tumor patients could not be ruled out. In the future, large and well-designed studies are required to illustrate the interactions of *VDR* genetic variants including *Fok I* polymorphism, environmental factors, life style and PCa.

## MATERIALS AND METHODS

### Literature and search strategy

The PubMed, Embase, Wanfang and Chinese National Knowledge Infrastructure (CNKI) database searches were conducted for all the eligible papers. The following search terms were used: “*VDR*/vitamin D receptor” and “prostate cancer/tumor/carcinoma”. Manually searching for the additional studies was conducted according to the references of the original and review reports. The literature search was updated on February, 2016.

### Study selection

Retrieved studies screened should meet the following criteria: (i) studies on human beings; (ii) in a case-control or nested case-control design; (iii) investigated the association between *VDR gene Fok I* polymorphism and PCa risk; (iv) detailed genotype distribution frequency of cases and controls could be obtained or calculated; (v) and received more than four points in the Newcastle-Ottawa Scale (NOS), which was considered to be high quality.

### Data extraction

The studies meeting the inclusion criteria were read carefully by two investigators independently (Yansheng Zhao and Lei Wang). The following information was extracted for reaching consensus on all of the items: the first author's name, year of publication, country of origin, ethnicity of study population, genotyping methods, source of controls, and number of cases and controls. The subjects were categorized as Asians, African and Caucasians for ethnicity; TaqMan, PCR-RFLP, SNPlex and other subgroup for genotyping method; population-based, hospital-based and Benign Prostate Hyperplasia (BPH) for the source of controls, respectively. We also divided the clinical stages into a localized group and an advanced group. Any disagreements were resolved by a third reviewer (Geng Zhao).

### Statistical analysis

A χ^2^-test based on the Q statistic was conducted to assess the heterogeneity. The between-study heterogeneity was considered to be significant when I^2^>50% and p<0.1, and the random effects model was chosen to combine values from studies [11]. Otherwise, for homogeneous studies, the fixed effects model was used. The pooled odds ratios (ORs) together with its 95% confidence intervals (95% CIs) were calculated to evaluate the risk. In addition, subgroup analyses were conducted based on ethnicity, genotyping method, source of controls and clinic stages. Sensitivity analysis was performed to assess the stability of pooled results. Begg's Funnel plot and Egger's test were preformed to assess the potential publication bias. Moreover, Hardy-Weinberg equilibrium (HWE) of controls was reexamined by us with the goodness-of-fit χ^2^-test. All analyses were performed using STATA package version 11.0 (Stata Corp, College Station, TX, USA).
